# A Novel Resource Polymorphism in Fish, Driven by Differential Bottom Environments: An Example from an Ancient Lake in Japan

**DOI:** 10.1371/journal.pone.0017430

**Published:** 2011-02-28

**Authors:** Takefumi Komiya, Sari Fujita, Katsutoshi Watanabe

**Affiliations:** Graduate School of Science, Kyoto University, Kyoto, Japan; University of Konstanz, Germany

## Abstract

Divergent natural selection rooted in differential resource use can generate and maintain intraspecific eco-morphological divergence (i.e., resource polymorphism), ultimately leading to population splitting and speciation. Differing bottom environments create lake habitats with different benthos communities, which may cause selection in benthivorous fishes. Here, we document the nature of eco-morphological and genetic divergence among local populations of the Japanese gudgeon *Sarcocheilichthys* (Cyprinidae), which inhabits contrasting habitats in the littoral zones (rocky vs. pebbly habitats) in Lake Biwa, a representative ancient lake in East Asia. Eco-morphological analyses revealed that *Sarcocheilichthys variegatus microoculus* from rocky and pebbly zones differed in morphology and diet, and that populations from rocky environments had longer heads and deeper bodies, which are expected to be advantageous for capturing cryptic and/or attached prey in structurally complex, rocky habitats. *Sarcocheilichthys biwaensis*, a rock-dwelling specialist, exhibited similar morphologies to the sympatric congener, *S. v. microoculus*, except for body/fin coloration. Genetic analyses based on mitochondrial and nuclear microsatellite DNA data revealed no clear genetic differentiation among local populations within/between the gudgeon species. Although the morphogenetic factors that contribute to morphological divergence remain unclear, our results suggest that the gudgeon populations in Lake Biwa show a state of resource polymorphism associated with differences in the bottom environment. This is a novel example of resource polymorphism in fish within an Asian ancient lake, emphasizing the importance and generality of feeding adaptation as an evolutionary mechanism that generates morphological diversification.

## Introduction

Resource polymorphism, the occurrence of intraspecific morphs within a single population exhibiting different niche use, is widespread over several taxa including fish, amphibians, and birds [1]. Resource polymorphism may be induced by phenotypic plasticity, genetic difference, or a combination of both. The mechanisms that generate and maintain the polymorphisms provide insights into the role of natural selection driving phenotypic, behavioral, and life-history diversification, which ultimately lead to speciation [1], [2].

Differences in prey utilization between structurally contrasting habitats often drive selection pressure for resource polymorphisms. Fish exhibit a variety of examples of resource polymorphisms, the majority of which include open-water habitats and the littoral zones of lakes, in which planktivorous limnetic and benthivorous benthic pairs often occur (e.g., three-spine stickleback [3]; Arctic charr [4]; bluegill sunfish [5]; Eurasian perch [6]). In typical cases, limnetic morphs exhibit a slim body that is well suited for cruising. Limnetics also have a larger number of gill rakers than do benthics, which is associated with a higher efficiency for capturing plankton. On the other hand, benthic morphs exhibit deep bodies, which enhance maneuverability. The large gapes of benthics are likely advantageous for suction feeding. Although rare, other pairs of resource morphs include, for example, littoral and profundal morphs in Arctic charr of Fjellfrøsvatn, north Norway [7] and snail-eating and algae-eating morphs in a cichlid fish of Cuatro Ciénegas, Mexico [8]. These dichotomies highlight the importance and generality of feeding adaptation in response to divergent natural selection that has arisen from environmental differences.

Here we examined another possible selection pressure that can promote resource polymorphism in fish in lake environments: selection derived from differential composition of bottom substrates. In lakes, habitats with differing bottom substrates harbor different benthic animals, as the physical and chemical characteristics of substrates strongly influence the structure of the benthos community [9]. For example, in comparison with structurally simple sandy or pebbly environments, a rocky environment offers many crevices that can serve as effective refuge places for particular taxa. Therefore, as bottom environments vary, so will the benthic communities they support. The diversity of benthos communities among habitats may generate divergent selection, favoring diverse feeding modes in benthivorous fish. These selection pressures are likely to promote resource polymorphism among populations that inhabit different bottom environments. So far, few examples of such resource morphs have been reported (e.g., Icelandic threespine sticklebacks between mud and lava habitats [10], [11]).

Lake Biwa, central Japan, is an ancient lake that is estimated to date back more than four million years [12]–[14], and the largest lake in Japan (surface area 670 km^2^; mean and maximum depth, 41 and 104 m, respectively). Since it first formed, the geological and limnological features of the lake have changed due to tectonic movements. The variety of habitats in Lake Biwa, which are characterized by a large and deep pelagic zone as well as complicated bottom environments in the littoral area, including sandy, pebbly, and rocky zones, were established in the mid-Pleistocene (ca. 0.4 million years ago [15]). The lake harbors 15 endemic fish species/subspecies that have evolved unique lifestyles suited to their respective habitats [16]–[18].

One group of such fishes, the gudgeon *Sarcocheilichthys* (Family Cyprinidae), provides an ideal opportunity for testing the hypothesis that bottom environmental differences lead to the evolution of resource polymorphism. *Sarcocheilichthys* usually feed on benthic prey from substrates by suction, swimming biased to the subbenthic column. These fish occur almost entirely in the littoral area of Lake Biwa. Thus, they utilize several types of bottom environments. Interestingly, previous studies noted that the gudgeons have a remarkable, continuous variation in head shape, exhibiting a short, intermediate, or long head [19]–[21]. Head shape divergence is often associated with diverse feeding modes in fishes [1]. Therefore, a detailed analysis of *Sarcocheilichthys* in Lake Biwa could provide another novel example of resource polymorphism.

Focusing on differences in bottom environments (rocky versus pebbly habitats), we assessed the genetic population structure and eco-morphological divergence among *Sarcocheilichthys* in Lake Biwa. First, based on mitochondrial DNA sequence and microsatellite data, we evaluated the degree of genetic differentiation among local populations. Second, using a geometric morphometrics technique, we tested the morphological divergence among local populations with respect to head and body shape, which are crucial for prey capture and locomotion in fish. Third, we analyzed the stomach contents of fishes from rocky and pebbly habitats. If divergent selection arising from prey use of different bottom environments is as strong as that displayed by the limnetic–benthic divergence, it would be reasonable to suppose eco-morphological divergence between the habitat types (i.e., resource polymorphism). In such a case, information regarding the genetic differentiation/structure at the local population level will help to elucidate the origin of the polymorphism and speciation process of this fish group.

## Materials and Methods

### Study species and sampling design

The study complies with the Fisheries Act in Japan, and was conducted under permission for fish sampling in Lake Biwa (#14-36 and #15-14) from the local government (Shiga Prefecture).

Two species/subspecies of *Sarcocheilichthys* endemically inhabit Lake Biwa, with different body colors and distributions [20], [21]. *Sarcocheilichthys variegatus microoculus* is an endemic form of *S. variegatus* that is distributed widely in western Japan and the Korean Peninsula. It has a grayish body and occurs throughout the littoral zone, including sandy, pebbly, and rocky zones. *Sarcocheilichthys biwaensis*, with a brownish body, strictly inhabits areas in and around rocky zones in the lake. The variation in head morphology shows a contrasting pattern between the two; the former exhibits large continuous variation, having a short, intermediate, or long head, whereas the latter has a long head [19]–[21]. These gudgeons exhibit similar characteristics in their gill rakers (coarse and fewer than 10), representing typical features of benthivores [20], [22], [23]. To date, no study has examined the genetic divergence and phylogenetic relationship of these fishes.

**Figure 1:**
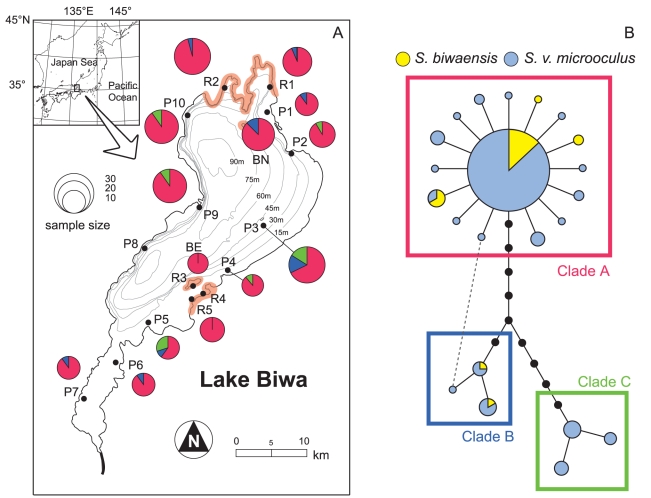
Sampling localities and mtDNA haplotype group frequencies of *Sarcocheilichthys* in Lake Biwa, Japan (A). Northern and eastern rocky zones are shaded in orange. **Statistical parsimony network for mtDNA of** ***Sarcocheilichthys*** **(B).** The areas of the circles are proportional to haplotype frequency. A dashed line indicates an alternative connection (loop). In both (A) and (B), each clade is shown in the same color. Sample codes correspond to those in Table 1.

For the morphometric and molecular analyses, we collected samples of *S. biwaensis* and *S. v. microoculus* from the entire shoreline of Lake Biwa between 2002 and 2007 (Figure 1A; Table 1). We identified the two groups primarily based on body color [20], [21]. Specimens from the collection at the Lake Biwa Museum, Shiga Prefecture, Japan, caught between 1974 and 1993, were also included among our samples. The total numbers of samples used in morphometric analyses were 508 and 370 from a total of 15 sites for head and body shape analyses, respectively, whereas for the molecular analyses, we used 207 and 181 samples from a total of 14 sites for the mtDNA and microsatellite analyses, respectively (see Table 1 for details). These local samples were, for convenience, regarded as “local populations.” Five rocky and ten pebbly local populations of *S. v. microoculus* were labeled using the sample codes R1–5 and P1–10, respectively. For *S. biwaensis*, because of the small sample size per site, samples from northern (R1–2, exceptionally captured from P1 and P10) and eastern (R3 and R5) rocky zones were pooled under the codes BN and BE, respectively. We judged whether each site represented a rocky or pebbly environment according to quantitative data on substratum constitutions reported by Nishino [24]; rocky habitats are restricted in relatively small areas of northern and eastern parts (Figure 1A).

**Table 1:** Species, habitat type (HT), sample code (SC), location, sampling year, and sample size for specimens used in this study.

Species	HT	SC	Location	Sampling year	Sample size		
					Morphology	mtDNA	Microsatellite
*S. biwaensis*	Rocky	BN	Northern rocky zone*	1974†, 1977†, 1990s–2006††, 2002, 2006	11 (11)	20	20
		BE	Eastern rocky zone**	No data, 1990s–2006††	11 (9)	7	8
*S. v. microoculus*	Rocky	R1	Kinomoto	2006	31 (31)	16	22
		R2	Oura	1978†, 2006	25 (23)	25	–
		R3	Okishima	2007	13 (9)	–	9
		R4	Miyagahama	1992†, 1993†	35 (28)	–	–
		R5	Mizugahama	2007	26 (22)	11	16
	Pebbly	P1	Onoe	2002	45 (25)	10	10
		P2	Minamihama	2003	16 (15)	12	–
		P3	Takeshima	2006	22 (7)	25	22
		P4	Notogawa	2002	33 (20)	9	–
		P5	Chuzu	2002	35 (28)	10	9
		P6	Moriyama	2002	54 (24)	10	8
		P7	Otsu	2002	41 (20)	10	10
		P8	Kitakomatsu	2004	25 (25)	–	15
		P9	Kitafunaki	2006	35 (25)	21	16
		P10	Momose	2006	50 (48)	21	16

The “Morphology” column lists sample sizes for heads and bodies (in parentheses).

*Pooled samples from Kinomoto (R1) and Oura (R2).

**Pooled samples from Okishima (R3) and Miyagahama (R4).

† Specimens deposited in Lake Biwa Museum (used for morphometric analysis).

†† Captive fish kept in Lake Biwa Museum (used for molecular analysis).

### Molecular analyses

A mitochondrial DNA sequence (cytochrome *b* gene) and 14 microsatellite loci were used to assess the genetic divergence among the local populations of *Sarcocheilichthys* in Lake Biwa. Genomic DNA was extracted from fin clips preserved in 100% ethanol using an Aqua Pure Genomic DNA Isolation Kit (Bio-Rad, Hercules, CA) according to the instructions provided by the manufacturer. For the mtDNA analysis, a partial sequence of the mtDNA cytochrome *b* (cyt*b*) region was amplified by polymerase chain reaction (PCR) using the primer pair L14724 (5′-TGACTTGAARAACCAYCGYYG-3′) [25] and H15915 (5′-ACCTCCGATCTYCGGATTACAAGAC-3′) [26]. PCR was carried out with a PC-808 thermal cycler (ASTEC, Fukuoka, Japan) using the following cycling program: 30 cycles of denaturation (94°C, 15 s), annealing (48°C, 15 s), and extension (72°C, 30 s). After purifying the PCR products by treatment with ExoSAP-It (usb Corp., Cleveland, OH) at 37°C, they were sequenced on an automated DNA sequencer (ABI Prism GA310; Applied Biosystems, Foster City, CA) with amplification primer H15915, using the BigDye Terminator Cycle Sequencing FS Ready Reaction Kit Ver. 1.1 (Applied Biosciences). The 3′-half of cyt*b* sequences (620 bp) were determined and deposited in the DDBJ, EMBL, and GenBank (accession numbers AB601449–601470). The haplotype frequency of each local population was deposited in GEDIMAP (P1279–1290, 1292–1296 [27]).

A total of 14 microsatellite loci isolated from *S. v. microoculus* [26] were analyzed, including 13 dinucleotide repeats (Svm03, Svm10, Svm32, Svm34, Svm46, Svm48, Svm49, Svm50, Svm53, Svm56, Svm72, Svm82, Svm166) and one trinucleotide repeat (Svm51). PCR conditions for each locus are described in Fujita et al. [28]. PCR products were sized on an automated DNA sequencer (ABI Prism GA310; Applied Biosystems, Foster City, CA) using GeneScan version 3.1 (ABI) and ROX400HD as the size standard (ABI).

Genetic divergence and structure among local populations were examined based on mtDNA and microsatellite data. To test the partitioning of genetic variation, we performed an analysis of molecular variance (AMOVA). AMOVA tests were conducted separately for species or habitat grouping to investigate inter- and intraspecific divergence. We further calculated the pairwise *F*_ST_ between local populations and genetic diversity indices as follows: number of haplotypes (*n*_h_), haplotype diversity (*h*) and nucleotide diversity (*π*) for mtDNA; mean number of alleles per locus (*n*_a_), allelic richness, mean observed (*H*_O_), and mean expected (*H*_E_) heterozygosities for microsatellites. These analyses were carried out using ARLEQUIN version 3.11 [29]. A Bayesian clustering approach, implemented using STRUCTURE version 2.3 software [30], was also used to estimate the population structure based on microsatellite data. We assumed the admixture model with correlated allele frequencies. Analyses were performed with a burn-in length of 30,000 and a run length of 300,000. Ten independent runs for each *K* (number of hypothetic genetic clusters, from 1 to 5) were evaluated, and ad hoc statistics (Δ*K*: second-order rate of change with respect to *K*) were calculated to determine the best estimation of *K* [31]. To infer population structures and historical processes resulting in the observed genetic distribution, a statistical parsimonious network was calculated for mtDNA haplotypes using the software TCS version 1.21 [32] at a 95% confidence limit.

### Morphometric analyses

**Figure 2:**
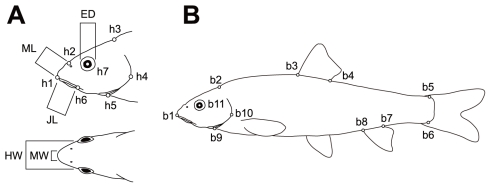
Landmarks used in morphometric analyses (A, B) and trophic traits (A). Trophic traits are eye diameter (ED), mouth length (ML), jaw length (JL), mouth width (MW), and head width (HW).

We hypothesized that natural selection might favor morphological divergence of multiple features (body units). We therefore focused on the relationships between locomotion behavior and body units that are often highlighted for successful feeding (e.g., the relationship between searching ability and body shape), as well as the relationship between prey-capture ability and head shape. To quantify shape variation among individuals, we conducted landmark-based geometric morphometric analyses (GM; [33], [34]). GM is a critical tool for analyzing morphometric shape variation. In contrast to the traditional linear measuring approach, GM retains the geometry among landmarks throughout the analysis, which makes it possible to generate graphical representations of shape variation. We first digitized the biologically homologous landmarks of each individual (Figure 2) on images using software TPSDIG2 version 2.12 [35]. For the alignment of the individuals, we used TPSREGR version 1.37 [36] to perform generalized procrustes superimposition [33], [37], which scales, translates, and rotates the landmarks to line them up as closely as possible. These superimposed landmarks were used to compute affine and non-affine shape components (i.e., uniform components and partial warps) using TPSREGR. We used these components as shape variables, drawing the morphology of each individual in the subsequent statistical analyses. The resulting shape axes were visualized as thin-plate spline transformation grids (constructed using TPSREGR), which exhibit lucid graphic display based on deformation grids [33].

We conducted a nested MANCOVA using shape variables (uniform components and partial warps) as a multivariate measure of shapes to test the morphological divergence between rocky and pebbly zones (i.e., habitat type) and among local populations nested within habitat type. Both *S. biwaensis* and *S. v. microoculus* local populations were included in the analysis. To control for differences in body size between populations, we used centroid size, which is defined as the square root of the sum of squared distances of all landmarks from their centroid, as a covariate. The above procedure returned canonical axes associated with habitat effect of each MANCOVA. To examine the nature of morphological divergence between habitats, we tested whether those canonical axes were correlated with superimposed landmark coordinates and also compared thin-plate spline transformation grids. Statistical analyses were conducted using JMP software version 5.01 (SAS Institute, Inc., Cary, NC). Discriminant function analyses (DFA) were performed using shape variables as the dependent factors and habitat type as the grouping factor. We examined how correctly individuals can be classified into their habitat type based on morphology. To enhance the reliability and generalizability of the classification, a cross-validation technique was included in our DFAs. DFAs were performed using SPSS version 17 (SPSS, Inc., Chicago, IL, USA).

A two-block partial least-squares analysis (PLS; [38]) was performed to assess the correlation between shape and trophic traits, i.e., eye diameter (ED), mouth length (ML), jaw length (JL), jaw width (JW), and head width (HW) (Figure 2A). PLS can explore patterns of covariation between two blocks of variables. PLS constructs pairs of variables that are linear combinations of the variables within each block. The linear combinations are formulated so that the new variables explain as much as possible of the covariation between the two original blocks of variables. Using these new variables, one can describe whatever patterns (i.e., dimensions) of covariation exist between the two blocks of original variables. Before PLS, the trophic traits and standard length were measured to the nearest 0.1 mm. Thereafter, each trait value was standardized according to the following formula, which reduces allometric effects on these traits [39].
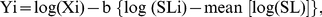
(1)where Yi and Xi are the adjusted and original values for the character in individual i (i = 1, . . . , N), SLi is the individual standard length, and b is the regression coefficient of the logarithm of X on the logarithm of SL. Significance of dimensions and correlations between blocks were computed using 1000 permutations. PLS was conducted using TPSPLS version 1.18 [40]. The pooled samples of *S. biwaensis* captured from the eastern rocky zone (BE) were excluded from the analysis due to poor measurement conditions.

### Diet analysis

Stomach contents of local populations of *S. v. microoculus* from two rocky (R4 and R5) and two pebbly (P2 and P8) sites were analyzed. Prey items were categorized under a stereoscope into six groups: chironomid and trichopteran larvae, snails, shrimps, zooplankton, and others. The relative contribution of each food group to the diet of an individual was estimated using the points method [41], a simple method that scores the relative volumes of each item. To quantify whether the gudgeons from different habitat types differed in diet, we conducted non-parametric MANOVA [42], [43] on Bray–Curtis distances for the proportions of prey groups with habitat type and local populations as factors, testing the significance by permuting the raw data (1000 permutations) using function adonis in the R package vegan version 1.17-4 [44]).

## Results

### Genetic population structure

A summary of the genetic diversity indices of mtDNA and microsatellites of each local population is presented in Table S1. A total of 22 mtDNA haplotypes were found in 207 sequences from *Sarcocheilichthys* in Lake Biwa, which were largely divided into three clades (A–C) in the network (Figure 1B). Clade A was the most frequent, the haplotypes of which were found within 182 individuals (87.9%) of all samples, with 151 (72.9%) individuals having the central haplotype. Clade A consisted of the single-step mutational haplotypes connected to the central one, showing a typical star-like structure, which is often found in populations having experienced a recent bottleneck [45]. The other two clades (B and C) were found at low frequencies, with 12 (5.8%) and 13 (6.3%) individuals, respectively. *Sarcocheilichthys biwaensis* shared haplotypes (clades A and B) largely with *S. v. microoculus*, with only two haplotypes being unique to the former.

The results of the AMOVAs based on both the mtDNA and microsatellite data showed a large amount of molecular variation within populations (>94% in all analyses) and low or no variation at the higher hierarchies (i.e., between species or habitat types, between populations within species/habitat types) (Table 2). There was significant, though slight (3.9%), molecular variance between habitats in mtDNA (*p* = 0.047). The pairwise *F*_ST_ tests among local populations showed non-significant values for all the comparisons in mtDNA, and all but two out of 78 microsatellite comparisons, for which significantly higher than zero (*p*<0.05) but quite low values (*F*_ST_ = 0.0447 and 0.0584) were represented (Table S2). In the Bayesian clustering analysis, the maximum value of Δ*K* was *K* = 2 (Δ*K* = 37.9; other Δ*K* values ranged from –1.2 to 15.3), but results showed no clear assignment bias among local populations (i.e., practically panmictic pattern; Figure S1 for the first supporting information figure). These results indicated that there was either very weak or no genetic population structuring among the gudgeon populations from different habitat types in Lake Biwa and even between *S. biwaensis* and *S. v. microoculus*.

**Table 2:** Analysis of molecular variance (AMOVA) for *Sarcocheilichthys* in Lake Biwa with (a) ‘species’ and (b) ‘habitat’ groupings.

Source of variation			DF	Sum of squares	Variance components	Percentage of variation	*F*-statistics	*p*
(a)	mtDNA	Between species	1	1.49	–0.005	–0.47	*F*_CT_ = –0.0047	0.384
		Between populations within species	12	19.46	0.042	4.03	*F*_SC_ = 0.0407	0.046
		Within populations	193	195.41	1.012	96.44	*F*_ST_ = 0.0356	0.032
	Microsatellite	Between species	1	6.86	0.005	0.1	*F*_CT_ = 0.0010	0.290
		Between populations within species	11	67.47	0.039	0.77	*F*_SC_ = 0.0077	<0.01
		Within populations	349	1769.29	5.070	99.13	*F*_ST_ = 0.0087	<0.001
(b)	mtDNA	Between habitats	1	5.45	0.042	3.9	*F*_CT_ = 0.0390	0.047
		Between populations within habitats	12	15.50	0.019	1.81	*F*_SC_ = 0.0189	0.207
		Within populations	193	195.41	1.012	94.28	*F*_ST_ = 0.0572	0.032
	Microsatellite	Between habitats	1	6.07	–0.002	–0.04	*F*_CT_ = –0.0004	0.434
		Between populations within habitats	11	68.26	0.042	0.82	*F*_SC_ = 0.0082	<0.001
		Within populations	349	1769.29	5.070	99.22	*F*_ST_ = 0.0078	<0.001

### Morphology

**Figure 3:**
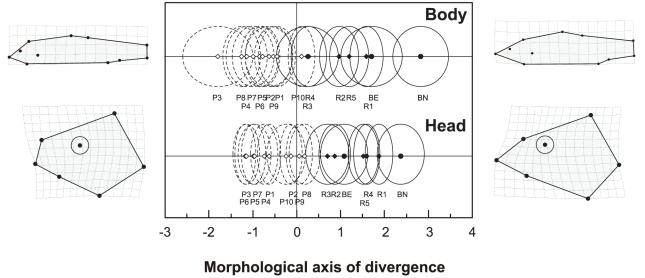
Morphological divergence between rocky and pebbly *Sarcocheilichthys* populations in Lake Biwa. The morphological index was derived from the MANCOVA for body and head shape, with thin-plate spline grids showing the shapes of individuals of plus and minus extremes (all magnified two times). For sample codes, see Figure 1A and Table 1.

The nested MANCOVAs revealed that habitat type had a significant effect on morphological divergence of the gudgeons in terms of both head and whole-body morphologies (Table 3). Head and body shape also changed under the influences of centroid size (i.e., multivariate allometry) and among local populations nested within habitat type. The effects of habitat type in the MANCOVAs explained a high degree of partial shape variance: 66.0% for head shape and 65.4% for body shape. Plots of population means on habitat canonical axes, along with thin-plate spline grids, are shown in Figure 3. We found significant correlations between the axes and superimposed landmark coordinates in both head and body (Table S3 and S4). These correlations and thin-plate spline grids provided interpretation of morphological divergence between habitats. Morphological shifts in rocky fish were: (1) an elongated head, longer mouth, and larger jaw in head shape, and (2) a longer head and a deeper and laterally more compressed body in body shape. Cross-validation included in DFAs revealed habitat-associated shape divergence among gudgeon populations (for head shape, Wilks' lambda = 0.517, *p*<0.001, and for body shape, Wilks' lambda = 0.634, *p*<0.001). The DFAs correctly assigned a majority of individuals to their respective habitats: 83.9% and 74.3% for head and body analysis, respectively (Table 3).

**Table 3:** Results of the multivariate analysis of covariance (MANCOVA) that tested for the effects of centroid size, habitat type, and population nested within habitat type on the head and body shape of *Sarcocheilichthys* in Lake Biwa.

Shape	*n*	Centroid size			Habitat type			Population (habitat type)			DFA results
		*F* Value	DF	*p*	*F* Value	DF	*p*	*F* Value	DF	*p*	
Head	508	8.7	10, 481	<0.0001	37.05	10, 481	<0.0001	9.24	150, 4051.2	<0.0001	83.9%
Body	370	4.43	18, 335	<0.0001	13.91	18, 335	<0.0001	5.77	270, 3917.5	<0.0001	74.3%

Results of discriminant function analysis (DFA) show percentages of individuals classified correctly into their respective habitats.

**Figure 4:**
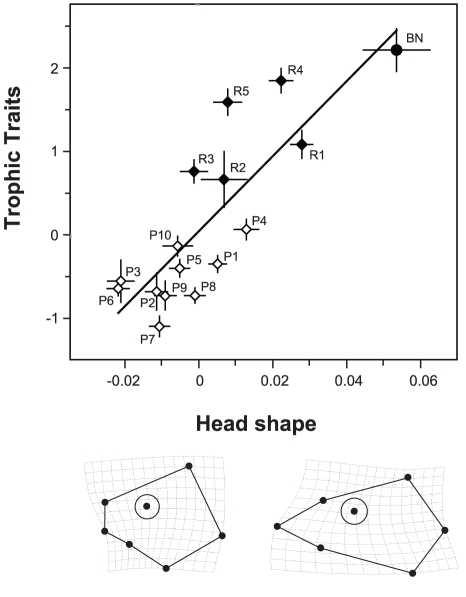
Correlation of variations between head shape and trophic traits revealed by PLS analysis. Individuals were pooled for local populations, with bars showing standard deviations. The coefficients of each trophic trait for “trophic traits” axis were the following: ED, 0.498; ML, 0.339; JL, 0.373; MW, 0.129; HW, –0.694. Thin-plate spline grids represent the shape of individuals of plus and minus extremes (all magnified two times). Sample codes correspond to those in Figure 1A and Table 1.

PLS yielded five dimensions of covariation between the two blocks of variable sets (i.e., head shape variables and trophic traits), of which only the first dimension was significantly greater than expected by chance (*p*<0.05). The first dimension accounted for 79.8% of the covariance. The correlation between the blocks was 0.52, which was also significant (*p*<0.01; Figure 4). On both the “head shape” and “trophic traits” axes, local populations were distributed so that the difference in habitat type served as a threshold. Based on thin-plate spline grids, the shape divergence axis represented a shape change for a short-headed (minus extreme) to a long-headed individual (plus extreme). We also estimated the coefficient of each trophic trait against the “trophic traits” axis: ED, 0.498; ML, 0.339; JL, 0.373; JW, 0.129; HW, –0.694. These results suggested that long-headed individuals, which were found at high frequencies in the rocky zone, have larger eyes, a longer mouth with a larger jaw, and a horizontally compressed head.

### Diet

**Figure 5:**
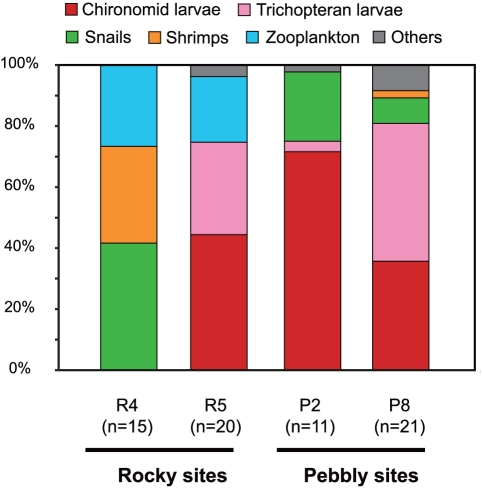
Stomach contents of *Sarcocheilichthys variegatus microoculus* captured in rocky (R4, R5) and pebbly zones (P2, P8), evaluated using Hynes's points method [**41**]. Sample codes correspond to those in Figure 1A and Table 1.

The non-parametric MANOVA revealed that the stomach contents of gudgeons were significantly different between habitat types and local populations (Figure 5, Table 4). The use of zooplankton such as *Daphnia* spp. was observed in moderate proportions in both rocky populations (26.7 and 21.6% in R4 and R5, respectively), whereas this was not the case for fishes from pebbly zones (P2 and P8). Although the proportions of the other prey groups were found in a mosaic fashion among the four local populations, the diet of the pebbly fish showed relatively similar composition, comprising mainly snails, chironomid, and trichopteran larvae. Fish from both the rocky (R5) and pebbly populations (P2 and P8) fed on trichopteran larvae, but used different ecological/taxonomic groups; i.e., fishes from R5 used mainly the larvae of the Polycentropodidae family, which makes cryptic scaffolding nets on the rock surface for filtering, whereas fishes from P2 and P8 fed mainly on larvae of the Leptoceridae, Hydroptilidae, and Sericostomatidae families, which are in portable organic or mineral cases and crawl around for filtering or gathering.

**Table 4:** Results of the non-parametric multivariate analysis of covariance (MANCOVA) for the stomach contents of *Sarcocheilichthys* from rocky and pebbly habitats.

Source	DF	Sum of squares	Mean squares	*F* Value	*p*
Habitat type	1	1.39	1.39	6.90	0.001
Local population	2	5.78	2.89	14.33	0.001
Residuals	63	12.71	0.20		
Total	66	19.88			

## Discussion

### Morphological differences between habitat types

*Sarcocheilichthys* usually swim close to the bottom (subbenthic habitat), searching for prey and picking it up from the substrates by suction [21; Komiya, personal observation]. We hypothesized that the fish forms responsible for prey capture (i.e., locomotion and suction feeding) would vary between populations inhabiting different bottom environments, i.e., rocky vs. pebbly zones. Indeed, the observed pattern of their morphological divergence largely matched the general view concerning the relationship between fish form and habitat complexity. Namely, in pebbly zones, where structurally simpler environments are widespread, gudgeon individuals were found to have a streamlined body with a short, round head, which would be optimal for minimizing water resistance (hence energy loss) during fast and extensive cruising while searching for widely dispersed prey [5], [46]–[49]. On the other hand, in the rocky zone, which provides a more complex bottom environment than a pebbly zone, we found that individuals had a deep and laterally compressed body with a long head. This is likely an adaptation for lower search velocities and high maneuverability, and for stronger suction ability for attacking prey [5], [46]–[50]. Moreover, we found a correlation between head shape and respective trophic traits. Longer-headed individuals from rocky zones were equipped with specialized traits, such as a narrow head, elongated mouth, large jaw, and large, anteriorly positioned eyes. All of these variations would be well suited to handling attached and/or cryptic prey hidden in crevices in the bottom or floating in open water (e.g., snails, caddisflies in scaffolding nets, shrimps, and zooplankton) [51]–[53]. Indeed, such prey items were found in fish from the rocky environment. Utilization of zooplankton is a common feature in limnetic morphs in several fish groups [1], [54]. However, this is not the case in *Sarcocheilichthys* in Lake Biwa; these gudgeons are typical suction feeders (not ram or filter feeders), and the degree of specialization to zooplankton seems to be low, as no divergence was observed in the number/density of gill rakers, which function as filters to capture small, plankton-like particles [22], [23]. Unfortunately, we did not directly compare benthos communities between rocky and pebbly habitats, and thus both the potential availability of each prey item and fish selectivity for specific items remain unknown. However, an array of comparative morphological and functional evidence strongly suggests the adaptive consequence of maximizing the efficiency of prey capture according to habitat. Namely, the morphological variation in the Lake Biwa gudgeons should be considered an example of resource polymorphism associated with the different bottom environments that developed in the ancient lake.

Other than the trophic traits around the buccal region (i.e., length of mouth and jaw, and width of mouth and head) that are clearly important for feeding adaptation, an alternative hypothesis for phenotypic differences corresponding to habitat type may involve divergent selection driven by differing predation threats among habitats. Traits that enhance the ability to escape would evolve with strong predation (e.g., [55]–[58]). For example, populations of mosquitofish (*Gambusia*) in the southern US and Caribbean islands have a highly developed caudal peduncle region that increases burst swimming speed when they co-occur with predatory fishes [59], [60]. In our case, rocky-habitat individuals had similarly deep bodies and caudal peduncles, which would enhance maneuverability and burst-swimming performance, as well as large eyes, which could be advantageous for detecting predators. In addition, the brownish body color of *S. biwaensis* might act as camouflage. These characteristics seem to be adapted to avoiding predation by piscivores such as the rock-dwelling catfish *Silurus lithophilus* and the cyprinid *Opsariichthys uncirostris,* which are endemic or semi-endemic species in Lake Biwa. Interestingly, few piscivorous fishes originally inhabited the shallow, pebbly zones of Lake Biwa (except for the largemouth bass, *Micropterus salmoides*, which invaded the lake a few decades ago), suggesting a higher predation threat in the rocky zone compared with the pebbly zone. As the feeding and anti-predation adaptation hypotheses do not contradict one another (i.e., similar morphological divergence would be predicted by both hypotheses), the synergetic effects of natural selection might have caused the observed body shape/color differences. Exploring the interaction between feeding adaptation and predation avoidance represents an interesting field for future studies.

### Genetic population structure and basis of resource polymorphism

Despite eco-morphological divergence, no evidence of clear population divergence among *Sarcocheilichthys* in Lake Biwa was obtained from either mitochondrial or nuclear genetic markers. Specifically, no genetic differentiation was detected within species from different habitat types or between *S. biwaensis* and *S. v. microoculus*. The panmictic status suggested by presumably neutral genetic markers may be due to recurrent moderate gene flow between populations/species. On the other hand, the typical star-like network shown for the major mtDNA clade (A) strongly implies a recent bottleneck in gudgeon populations [45]. A bottleneck event, in general, causes a decrease in the genetic diversity of a population and might result in an underestimation of population divergence if random fixation into common major haplotypes tended to occur. Also, if the bottleneck occurred recently, there would have been insufficient time to accumulate genetic differences in neutral markers, even though some reproductive barrier exists between populations or species. A weak (3.9%) but significant habitat effect on molecular variance in mtDNA may be a footprint of genetic divergence before or after the bottleneck event.

No differentiation signal was found between *S. v. microoculus* and *S. biwaensis* even in hyper-variable microsatellite markers. They mostly shared the mtDNA haplotypes of the major clade (A) and one of the two minor clades (B). This suggests three possibilities. The first is that *S. biwaensis* is just a color variant of *S. v. microoculus*, which is strictly restricted to the major rocky areas, possibly associated with some local adaptation. *Sarcocheilichthys biwaensis* was somewhat recently described, mainly based on unique body/fin color, and its body and head shape largely overlap with those of the ‘long-headed’ type of *S. v. microoculus* from rocky areas [20; the present study]. The reproductive isolation between *S. biwaensis* and *S. v. microoculus* has not been well examined yet, although they do crossbreed in captivity [19; Komiya, personal observation]. The second possibility is that they have developed some reproductive isolation mechanism, and *S. biwaensis* is recently derived from *S. variagatus* ancestral stock, meaning that it has not yet accumulated genetic differences in any neutral markers.

The other possible reason for no interspecific genetic difference is historical and/or contemporary introgressive hybridization between two well-differentiated species. In addition to the original smaller population size of *S. biwaensis* [20], [21], further population declines due to predation by piscivorous alien species, various human impacts, and possible hybridization with *S. v. microoculus* have been suggested (Japan Ministry of the Environment 2003). This situation implies the possibility of extinction of genetically pure *S. biwaensis*, even if it was present in the past. In any case, *S. biwaensis* was revealed to be a genetically indefinable form, at least at present. Further research is needed to examine the reproductive isolation between the two species under natural conditions.

The phenotypic divergence observed in *Sarcocheilichthys* in Lake Biwa could be caused by phenotypic plasticity, genetic differences, or a combination of both. In general, the relative importance of these effects varies among fish species [1], [54]. To date, there are no data to indicate how strongly each factor may be able to influence the observed morphological differences in *Sarcocheilichthys*. However, according to Nakamura's [19] brief description, body color is inherited in a Mendelian fashion, with the brownish color of *S. biwaensis* being recessive. Also noted was that head-length variation was somewhat genetically controlled. The existence of genetic components in these morphologies suggests adaptive genetic divergence between populations utilizing different resources. Although the panmictic status of the Lake Biwa gudgeons was revealed by presumably neutral markers, and extensive gene flow was expected among them, it is possible that only specific genes associated with local adaptation show differentiation between populations/species [61]–[63]. Further population genetic analyses using such adaptation-related genes (e.g., those associated with body color and head length/shape, as candidates) will contribute to our knowledge of ecological speciation processes.

## Supporting Information

Figure S1 **Bayesian clustering analysis of** ***Sarcocheilichthys*** **populations using STRUCTURE** [**30**] **for** ***K*** **= 2.** Each vertical bar represents an individual partitioned into the two clusters defined by STRUCTURE. Sample codes correspond to those in Figure 1A and Table 1.
(EPS)

Table S1 Genetic diversity estimates for *Sarcocheilichthys* in Lake Biwa: SC, sample codes; *n*, number of samples; *n_h_*, number of haplotypes; *h*, haplotype diversity; *π*, nucleotide diversity; *n*_a_, number of alleles; *AR*, allelic richness; *H_O_*, observed heterozygosity; *H_E_*, expected heterozygosity. For microsatellites, no excesses or deficits in heterozygosity were found among any local samples (*α* = 0.05).
(DOC)

Table S2 Pairwise *F*_ST_ values between local populations (SC, sample codes): above diagonal, microsatellite; below diagonal, mtDNA.
(DOC)

Table S3 Correlation between superimposed landmark coordinates of head shape and the habitat canonical axis from the MANCOVA. Significant values are shown in bold (*p*<0.05). The final column shows the relative direction of landmarks found in rocky populations compared to pebbly populations. For example, landmark h1 is located at a relatively anterior and dorsal position in rocky populations.
(DOC)

Table S4 Correlation between superimposed landmark coordinates of body shape and the habitat canonical axis from the multivariate analysis of covariance (MANCOVA). Significant values are shown in bold (*p*<0.05). The final column shows the relative direction of landmarks found in rocky populations compared to those of pebbly populations. For example, landmark b5 is located at a relatively anterior and dorsal position in rocky populations.
(DOC)
