# Supplementary material for: A Novel Resource Polymorphism in Fish, Driven by Differential Bottom Environments: An Example from an Ancient Lake in Japan
Source: PLoS One. 2011 Feb 28;6(2):e17430. doi: 10.1371/journal.pone.0017430 (PMC3046152; doi:10.1371/journal.pone.0017430)
Supplement: Table S1 — Genetic diversity estimates for Sarcocheilichthys in Lake Biwa: SC, sample codes; n, number of samples; nh, number of haplotypes; h, haplotype diversity; π, nucleotide diversity; n a, number of alleles; AR, allelic richness; HO, observed heterozygosity; HE, expected heterozygosity. For microsatellites, no excesses or deficits in heterozygosity were found among any local samples (α = 0.05). (DOC) [file pone.0017430.s002.doc]

**Table S1.** Genetic diversity estimates for *Sarcocheilichthys* in Lake Biwa: SC, sample codes; *n*, number of samples; *n_h_*, number of haplotypes; *h*, haplotype diversity; *π*, nucleotide diversity; *n*_a_, number of alleles; *AR*, allelic richness; *H_O_*, observed heterozygosity; *H_E_*, expected heterozygosity. For microsatellites, no excesses or deficits in heterozygosity were found among any local samples (*α* = 0.05).

| **SC** | **Location** | **mtDNA sequence** | | | | **Microsatellite** | | | | |
| --- | --- | --- | --- | --- | --- | --- | --- | --- | --- | --- |
|  |  | ***n*** | ***n_h_*** | ***h* (± SD)** | ***π* (± SD) × 10^–2^** | ***n*** | ***n_a_* (± SD)** | ***AR*** | ***H_O_* (± SD)** | ***H_E_* (± SD)** |
| BN | Northern rocky zone* | 20 | 6 | 0.516 (0.132) | 0.262 (0.181) | 20 | 9.14 (4.02) | 6.27 | 0.743 (0.184) | 0.746 (0.170) |
| BE | Eastern  rocky zone** | 7 | 2 | 0.571 (0.120) | 0.092 (0.096) | 8 | 5.14 (2.28) | 5.14 | 0.679 (0.280) | 0.684 (0.193) |
| R1 | Kinomoto | 16 | 4 | 0.350 (0.148) | 0.182 (0.139) | 22 | 9.64 (4.77) | 6.49 | 0.716 (0.210) | 0.734 (0.189) |
| R2 | Oura | 25 | 5 | 0.300 (0.118) | 0.129 (0.107) | – | – | – | – | – |
| R3 | Okishima | – | – | – | – | 9 | 6.14 (2.93) | 6.32 | 0.746 (0.244) | 0.721 (0.213) |
| R5 | Mizugahama | 11 | 2 | 0.327 (0.153) | 0.053 (0.065) | 16 | 8.36 (4.53) | 6.14 | 0.683 (0.265) | 0.711 (0.232) |
| P1 | Onoe | 10 | 4 | 0.533 (0.180) | 0.258 (0.189) | 10 | 7.29 (3.02) | 6.10 | 0.686 (0.260) | 0.725 (0.201) |
| P2 | Minamihama | 12 | 4 | 0.455 (0.170) | 0.296 (0.205) | – | – | – | – | – |
| P3 | Takeshima | 25 | 8 | 0.640 (0.107) | 0.709 (0.403) | 22 | 9.93 (5.03) | 6.33 | 0.724 (0.233) | 0.727 (0.210) |
| P4 | Notogawa | 9 | 5 | 0.806 (0.120) | 0.493 (0.321) | – | – | – | – | – |
| P5 | Chuzu | 10 | 5 | 0.756 (0.130) | 0.882 (0.524) | 9 | 6.36 (2.56) | 6.27 | 0.698 (0.211) | 0.733 (0.198) |
| P6 | Moriyama | 10 | 3 | 0.378 (0.181) | 0.226 (0.171) | 8 | 5.64 (2.62) | 5.87 | 0.723 (0.209) | 0.723 (0.181) |
| P7 | Otsu | 10 | 3 | 0.378 (0.181) | 0.258 (0.189) | 10 | 7.14 (3.35) | 6.14 | 0.707 (0.246) | 0.709 (0.219) |
| P8 | Kitakomatsu | – | – | – | – | 15 | 8.07 (3.63) | 6.05 | 0.738 (0.208) | 0.738 (0.192) |
| P9 | Kitafunaki | 21 | 4 | 0.271 (0.124) | 0.309 (0.204) | 16 | 8.07 (4.25) | 5.64 | 0.723 (0.240) | 0.710 (0.236) |
| P10 | Momose | 21 | 5 | 0.424 (0.131) | 0.309 (0.204) | 16 | 8 (3.74) | 6.37 | 0.670 (0.234) | 0.722 (0.186) |

* Pooled samples from Kinomoto (R1) and Oura (R2).

** Pooled samples from Okishima (R3) and Mizugahama (R5).
