# Supplementary material for: A Novel Resource Polymorphism in Fish, Driven by Differential Bottom Environments: An Example from an Ancient Lake in Japan
Source: PLoS One. 2011 Feb 28;6(2):e17430. doi: 10.1371/journal.pone.0017430 (PMC3046152; doi:10.1371/journal.pone.0017430)
Supplement: Table S2 — Pairwise F ST values between local populations (SC, sample codes): above diagonal, microsatellite; below diagonal, mtDNA. (DOC) [file pone.0017430.s003.doc]

**Table S2.** Pairwise *F*_ST_ values between local populations (SC, sample codes): above diagonal, microsatellite; below diagonal, mtDNA.

| **SC** | **BN** | **BE** | **R1** | **R2** | **R3** | **R5** | **P1** | **P2** | **P3** | **P4** | **P5** | **P6** | **P7** | **P8** | **P9** | **P10** |
| --- | --- | --- | --- | --- | --- | --- | --- | --- | --- | --- | --- | --- | --- | --- | --- | --- |
| **BN** | – | 0.0246 | –0.0004 | – | –0.0009 | –0.0025 | –0.0058 | – | –0.0014 | – | –0.0041 | 0.0197 | 0.0125 | –0.0030 | 0.0087 | –0.0055 |
| **BE** | 0.0589 | – | 0.0330 | – | 0.0219 | 0.0303 | 0.0310 | – | **0.0447** | – | 0.0218 | 0.0581 | 0.0501 | 0.0333 | **0.0584** | 0.0342 |
| **R1** | –0.0375 | 0.0876 | – | – | 0.0079 | 0.0030 | 0.0022 | – | 0.0018 | – | –0.0015 | 0.0223 | 0.0004 | 0.0055 | 0.0037 | –0.0029 |
| **R2** | –0.0133 | 0.0974 | –0.0418 | – | – | – | – | – | – | – | – | – | – | – | – | – |
| **R3** | – | – | – | – | – | –0.0025 | 0.0019 | – | 0.0130 | – | –0.0139 | 0.0232 | 0.0036 | –0.0031 | 0.0191 | 0.0077 |
| **R5** | 0.0228 | 0.2860 | 0.0079 | 0.0113 | – | – | 0.0052 | – | 0.0031 | – | –0.0075 | 0.0161 | 0.0116 | 0.0051 | 0.0109 | –0.0040 |
| **P1** | –0.0581 | 0.0605 | –0.0639 | –0.0341 | – | 0.0254 | – | – | –0.0011 | – | –0.0073 | 0.0166 | 0.0194 | 0.0073 | 0.0188 | –0.0026 |
| **P2** | –0.0207 | 0.0223 | –0.0283 | –0.0107 | – | 0.0108 | –0.0467 | – | – | – | – | – | – | – | – | – |
| **P3** | 0.0643 | 0.1132 | 0.0781 | 0.1308 | – | 0.1198 | 0.0291 | 0.0323 | – | – | –0.0013 | 0.0203 | 0.0140 | 0.0095 | 0.0067 | –0.0036 |
| **P4** | 0.0048 | 0.0164 | –0.0115 | 0.0355 | – | 0.0465 | –0.0443 | –0.0632 | 0.0011 | – | – | – | – | – | – | – |
| **P5** | 0.1812 | 0.2037 | 0.2059 | 0.2937 | – | 0.2477 | 0.1211 | 0.0992 | –0.0374 | 0.0423 | – | 0.0146 | 0.0121 | –0.0039 | 0.0141 | –0.0013 |
| **P6** | –0.0608 | 0.1039 | –0.0601 | –0.0297 | – | 0.0272 | –0.0870 | –0.0449 | 0.0301 | –0.0241 | 0.1286 | – | 0.0116 | 0.0139 | 0.0315 | 0.0147 |
| **P7** | –0.0616 | 0.0913 | –0.0639 | –0.0299 | – | 0.0254 | –0.0811 | –0.0414 | 0.0250 | –0.0235 | 0.1211 | –0.0870 | – | 0.0183 | 0.0224 | 0.0129 |
| **P8** | – | – | – | – | – | – | – | – | – | – | – | – | – | – | 0.0152 | 0.0049 |
| **P9** | 0.0018 | 0.0692 | –0.0069 | 0.0139 | – | 0.0181 | –0.0291 | –0.0569 | 0.0436 | –0.0433 | 0.1186 | –0.0277 | –0.0236 | – | – | –0.0018 |
| **P10** | 0.0018 | 0.0692 | –0.0108 | 0.0139 | – | 0.0181 | –0.0291 | –0.0569 | 0.0436 | –0.0433 | 0.1186 | –0.0277 | –0.0236 | – | –0.0448 | – |

Sample codes correspond to those in Figure 1A and Tables 1 and S1.

Bold values are significant at the 5% level after sequential Bonferroni correction.
