# Supplementary material for: A Novel Resource Polymorphism in Fish, Driven by Differential Bottom Environments: An Example from an Ancient Lake in Japan
Source: PLoS One. 2011 Feb 28;6(2):e17430. doi: 10.1371/journal.pone.0017430 (PMC3046152; doi:10.1371/journal.pone.0017430)
Supplement: Table S3 — Correlation between superimposed landmark coordinates of head shape and the habitat canonical axis from the MANCOVA. Significant values are shown in bold (p<0.05). The final column shows the relative direction of landmarks found in rocky populations compared to pebbly populations. For example, landmark h1 is located at a relatively anterior and dorsal position in rocky populations. (DOC) [file pone.0017430.s004.doc]

**Table S3.** Correlation between superimposed landmark coordinates of head shape and the habitat canonical axis from the MANCOVA. Significant values are shown in bold (*p* < 0.05). The final column shows the relative direction of landmarks found in rocky populations compared to pebbly populations. For example, landmark h1 is located at a relatively anterior and dorsal position in rocky populations.

| **Landmark** | **Spearman's r** | **Direction** |
| --- | --- | --- |
| h1X | **–0.5009** | anterior |
| h1Y | **0.1916** | dorsal |
| h2X | **0.5840** | posterior |
| h2Y | **–0.0994** | ventral |
| h3X | 0.0261 | – |
| h3Y | **–0.2820** | ventral |
| h4X | **–0.2018** | anterior |
| h4Y | **0.1258** | dorsal |
| h5X | **0.3691** | posterior |
| h5Y | –0.0437 | – |
| h6X | **–0.2606** | anterior |
| h6Y | **0.1454** | dorsal |
| h7X | **–0.5467** | anterior |
| h7Y | –0.0723 | – |
