# Supplementary material for: A Novel Resource Polymorphism in Fish, Driven by Differential Bottom Environments: An Example from an Ancient Lake in Japan
Source: PLoS One. 2011 Feb 28;6(2):e17430. doi: 10.1371/journal.pone.0017430 (PMC3046152; doi:10.1371/journal.pone.0017430)
Supplement: Table S4 — Correlation between superimposed landmark coordinates of body shape and the habitat canonical axis from the multivariate analysis of covariance (MANCOVA). Significant values are shown in bold (p<0.05). The final column shows the relative direction of landmarks found in rocky populations compared to those of pebbly populations. For example, landmark b5 is located at a relatively anterior and dorsal position in rocky populations. (DOC) [file pone.0017430.s005.doc]

**Table S4.** Correlation between superimposed landmark coordinates of body shape and the habitat canonical axis from the multivariate analysis of covariance (MANCOVA). Significant values are shown in bold (*p* < 0.05). The final column shows the relative direction of landmarks found in rocky populations compared to those of pebbly populations. For example, landmark b5 is located at a relatively anterior and dorsal position in rocky populations.

| **Landmark** | **Spearman's r** | **Direction** |
| --- | --- | --- |
| b1X | **–0.4809** | anterior |
| b1Y | 0.0724 | – |
| b2X | **0.2481** | posterior |
| b2Y | 0.0514 | – |
| b3X | 0.0187 | – |
| b3Y | **0.1786** | dorsal |
| b4X | **0.3179** | posterior |
| b4Y | 0.0402 | – |
| b5X | **–0.2929** | anterior |
| b5Y | **0.2383** | dorsal |
| b6X | **–0.2283** | anterior |
| b6Y | 0.0412 | – |
| b7X | –0.0317 | – |
| b7Y | **–0.1952** | ventral |
| b8X | 0.0378 | – |
| b8Y | **–0.4140** | ventral |
| b9X | **0.3271** | posterior |
| b9Y | **–0.3381** | ventral |
| b10X | **0.2427** | posterior |
| b10Y | –0.0110 | – |
| b11X | **–0.5195** | anterior |
| b11Y | 0.0178 | – |
